# Liquid Biopsy Biomarkers for Predicting and Monitoring Immunotherapy Response in Lung Cancer

**DOI:** 10.3390/cancers18111840

**Published:** 2026-06-04

**Authors:** Viola Bianca Serio, Tommaso Regoli, Elisa Frullanti, Maria Palmieri

**Affiliations:** 1 Cancer Genomics & Systems Biology Lab, University of Siena, 53100 Siena, Italy; viola.serio@dbm.unisi.it (V.B.S.); tommaso.regoli@student.unisi.it (T.R.); maria.palmieri@dbm.unisi.it (M.P.); 2 Med Biotech Hub and Competence Centre, Department of Medical Biotechnologies, University of Siena, 53100 Siena, Italy; 3 Department of Medical, Surgical and Neurological Sciences, University of Siena, 53100 Siena, Italy

**Keywords:** lung cancer, liquid biopsy, immunotherapy, ctDNA, PD-L1, CTC, exosomes, tumor mutational burden, cytokines

## Abstract

**Background**: While Immune Checkpoint Inhibitors (ICIs) have significantly improved outcomes in lung cancer (LC), clinical responses remain heterogeneous. Static tissue biomarkers, like PD-L1, and tumor mutational burden (TMB) are limited by intratumoral heterogeneity and the inability to track temporal changes. This review aims to evaluate the current state and future potential of liquid biopsy as a dynamic tool for patient selection, treatment monitoring, and the identification of resistance mechanisms in LC immunotherapy. **Methods:** A literature search was conducted in the PubMed database up to March 2026. We identified 65 eligible publications, including clinical trials, observational studies, and systematic reviews, focusing on liquid biopsy analytes such as circulating tumor DNA (ctDNA), circulating tumor cells (CTCs), exosomes, and soluble immune mediators. **Results:** Liquid biopsy provides a “pooled” representation of the total tumor burden, overcoming the spatial limitations of tissue biopsy. Key findings include that dynamic changes in ctDNA and bTMB can predict molecular progression weeks before radiological assessment; blood-based PD-L1 monitoring (soluble, exosomal, or on CTCs) correlates with survival outcomes and offers a real-time readout of immune checkpoint activity; novel markers like tumor-macrophage fusion (TMF) cells and cytokine signatures provide unique insights into the systemic immune microenvironment. **Conclusions:** Liquid biopsy is evolving from a complementary diagnostic tool into a central pillar of precision immuno-oncology. Although technical standardization remains a challenge, the integration of multi-omic blood-based biomarkers represents the future of personalized lung cancer management.

## Simple Summary

Immunotherapy has revolutionized the treatment of lung cancer, but only a fraction of patients derive long-term benefit. Currently, tissue samples are used to predict treatment success, but these samples often fail to capture the complete and changing the nature of the entire tumor. This review explores “liquid biopsy”, a minimally invasive method to analyze cancer markers from blood, as a tool to better select patients and monitor their response to therapy in real time. We reported various blood-based markers data, such as circulating DNA and specialized immune cells, in order to understand whether a pharmacological treatment is working months before traditional scans. The collected data reported in this review suggest that liquid biopsy could soon complement or even replace traditional biopsies, offering a more precise and dynamic way to manage lung cancer treatment.

## 1. Introduction

In recent years, Immune Checkpoint Inhibitors (ICIs) targeting the PD-1/PD-L1 axis have revolutionized the treatment landscape of lung cancer (LC), leading to significant improvements in overall survival (OS) and progression-free survival (PFS) in a subset of patients [1,2]. Nevertheless, clinical responses remain markedly heterogeneous with only a fraction of individuals achieving durable benefit. This variability highlights the urgent need for reliable, dynamic, and minimally invasive biomarkers able to better predict and monitor therapeutic efficacy.

Against this backdrop, liquid biopsy has emerged as a transformative tool for the real-time molecular characterization of tumors and their evolving microenvironment. Since the initial conceptualization of analyzing circulating components—such as circulating tumor DNA (ctDNA), circulating tumor cells (CTCs), and extracellular vesicles—to non-invasively assess tumor mutational profiles [3], the field has rapidly evolved. Subsequent research expanded this foundation, demonstrating that liquid biopsy is not only pivotal for patient stratification in the context of immunotherapy but also essential for the early assessment of treatment response and the identification of resistance mechanisms to anti-PD-L1 therapies [4].

More recently, Yang et al. (2023) further delineated the evolving role of liquid biopsy as an integrative platform capable of combining genomic, epigenomic, and immunological information [5]. In particular, the dynamic monitoring of ctDNA, blood-based tumor mutational burden (bTMB), and DNA methylation patterns is demonstrating increasing potential in predicting response to PD-L1 inhibitors and in distinguishing true disease progression from pseudoprogression, which is one of the foremost clinical challenges in immunotherapy.

Despite these advances, significant technical and biological hurdles persist, including constraints related to assay sensitivity, tumor heterogeneity, and the lack of standardization across analytical platforms. Consequently, further prospective validation is warranted before liquid biopsy can be fully integrated into routine clinical decision making.

Although ICIs have shown clinical activity across multiple tumor types, their most extensive clinical development and biomarker integration have been achieved in non-small cell lung cancer (NSCLC), establishing it as a paradigmatic model for investigating response and resistance mechanisms as well as for the development and validation of predictive biomarkers to guide patient selection and optimize therapeutic strategies.

This review aims to provide a comprehensive overview of the current state of the art and future perspectives of liquid biopsy in the setting of anti-PD-L1 immunotherapy in LC with particular emphasis on its role in patient selection, treatment monitoring, and the elucidation of resistance mechanisms.

## 2. Materials and Methods

### Search Strategy, Inclusion Criteria and Data Collection

A systematic literature search was conducted in the PubMed database up to 23 March 2026 for publications reporting studies that focus on the benefit of liquid biopsy in immunotherapy management in lung cancer patients using the following keywords: “immunotherapy” AND” lung cancer” AND “ liquid biopsy”.

Filters were applied to include only articles with available full text and free full text. The search was restricted to studies conducted in humans and published in English.

Regarding study design, we included a broad range of article types to ensure comprehensive coverage of the available evidence. Specifically, the following categories were considered: clinical studies and clinical trials (including Phase I–IV trials and trial protocols), randomized controlled trials, controlled clinical trials, observational and multicenter studies, comparative and evaluation studies, validation studies, as well as case reports.

Furthermore, secondary research articles, such as systematic reviews, scoping reviews, meta-analyses, and network meta-analyses, were included. Clinical guidelines and practice guidelines were also considered to provide a broader clinical context. Datasets were included when relevant. No restrictions regarding patients’ sex or age were applied. No filters were applied to exclude preprints or to restrict results to MEDLINE-indexed articles.

**Figure 1:**
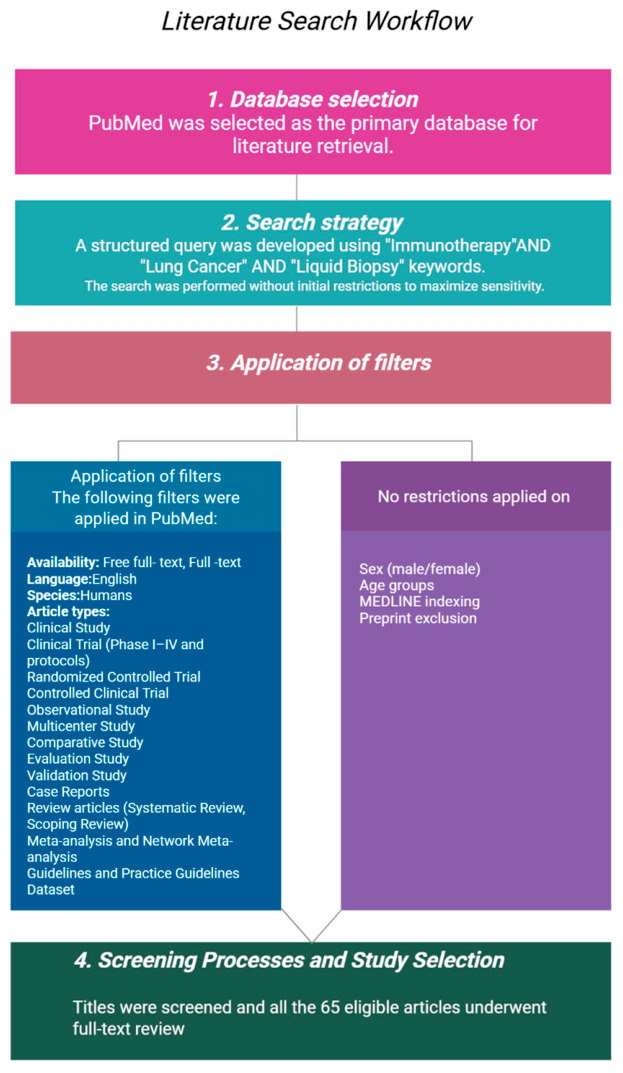
Literature search flow. Starting from the PubMed database, by using keywords and applying the listed specific filters, 65 eligible papers have been selected for being reviewed.

A total of 65 eligible publications (published from 2017 to March 2026) were identified and evaluated. Following initial retrieval of 65 candidate references through database searching, full-text screening was performed independently by the authors (V.B.S., E.F., M.P.). After the exclusion of articles that did not meet the predefined inclusion criteria (such as insufficient relevance to liquid biopsy in the context of immunotherapy for NSCLC), a final set of 57 references was retained and forms the evidence base of the present review. Any discrepancies during the screening of titles, abstracts, and full texts were resolved through inter-reviewer discussion until a consensus was reached. A flowchart of the literature selection is shown in Figure 1.

## 3. Results

### 3.1. Liquid Biopsy of Biomarkers for Immunotherapy

Liquid biopsy has emerged as a precision medicine tool in immuno-oncology, enabling the non-invasive detection and longitudinal monitoring of a broad spectrum of tumor-derived biomarkers from peripheral blood and other body fluids. Lung cancer (LC) remains the leading cause of cancer-related deaths worldwide, and the development of minimally invasive diagnostic tools has become a clinical priority given that most patients are diagnosed at advanced stages [5]. Liquid biopsy includes testing on a variety of cancer biomarkers, such as circulating tumor DNA (ctDNA), microRNA, and circulating tumor cells (CTCs), and being minimally invasive, it can be collected from plasma, serum, urine, cerebrospinal fluid (CSF), and other sources to determine actionable genomic alterations that may eventually guide therapy and help to assess response. A consensus statement from the International Association for the Study of Lung Cancer (IASLC) formally endorsed the clinical utility of liquid biopsy in advanced NSCLC, recognizing its role in molecular profiling, resistance monitoring, and complementing tissue-based analyses, particularly when tissue is insufficient or unavailable [6].

The development of reliable blood-based biomarkers for immunotherapy has become a major research priority, given that current tissue-based markers present significant limitations in detecting the dynamic and spatially heterogeneous nature of the tumor immune landscape [4,7,8]. The liquid biopsy analytes currently investigated in this context are circulating tumor cells, soluble checkpoint molecules, free nucleic acids including ctDNA, exosomes, and protein-based ECM (extracellular matrix) remodeling fragments. More recently, microparticles and extracellular vesicles have emerged as additional liquid biopsy substrates with diagnostic and prognostic potential in LC, carrying tumor-derived proteins, nucleic acids, and lipids that reflect the state of the tumor microenvironment [9,10].

**Figure 2:**
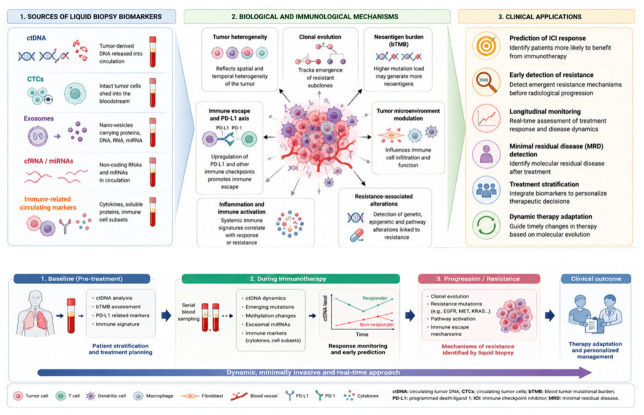
Biological framework and clinical integration of liquid biopsy biomarkers in lung cancer immunotherapy. This schematic presentation outlines the comprehensive role of liquid biopsy by connecting biomarker sources (ctDNA, CTCs, exosomes, cfRNA/miRNAs, and immune-related circulating markers) to their underlying biological and immunological mechanisms, such as tumor heterogeneity, clonal evolution, neoantigen burden, PD-1/PD-L1 axis immune escape, microenvironment modulation, and inflammation, while mapping them to key clinical applications including the prediction of immune checkpoint inhibitor (ICI) response. Furthermore, it provides a dynamic, longitudinal overview of how these biomarkers and their mechanistic pathways are integrated into practice across different clinical phases, spanning from baseline patient stratification and treatment planning to real-time response monitoring (e.g., via ctDNA dynamics) and the tracking of resistance mechanisms during disease progression.

Comprehensive reviews confirm that liquid biopsy holds promise not only as a companion diagnostic tool for immunotherapy but also as a monitoring instrument across the entire treatment trajectory, from early detection through to resistance profiling, with ongoing technical refinement needed to standardize platforms and interpretation frameworks [11,12,13,14,15]. Collectively, these liquid biopsy approaches hold the potential to complement and may replace tissue biopsy for guiding immunotherapy decisions, offering a real-time molecular portrait of the tumor immune landscape (Figure 2). 

### 3.2. Immunotherapy in Lung Cancer

Immunotherapy, particularly by using ICIs targeting the PD-1/PD-L1 axis, has fundamentally reshaped the therapeutic landscape of NSCLC. NSCLC represents approximately 85% of all LC diagnoses and encompasses several histological subtypes with distinct biological and molecular characteristics [16]. Immunotherapy has demonstrated a survival benefit in patients with locally advanced NSCLC. In patients with no targetable genetic alterations and no contraindications to PD-1/PD-L1 inhibitors, immunotherapy, either as monotherapy or in combination, has become the standard of care in the front-line setting for advanced squamous and non-squamous lung cancer. The pivotal *KEYNOTE-024* trial established pembrolizumab monotherapy as superior to platinum-based chemotherapy in patients with PD-L1 TPS (Tumor Proportion Score) (which represents the percentage of viable cells expressing PDL1) ≥50%, demonstrating a superior overall survival of 30.0 months versus 14.2 months. For patients with lower or absent PD-L1 expression, combination chemo-immunotherapy regimens have demonstrated consistent overall survival benefits, as shown by *KEYNOTE-189* and *KEYNOTE-407*. Beyond the advanced setting, in the PACIFIC trial, patients who received anti-PD-L1 durvalumab after chemoradiation had a remarkable improvement in overall survival with median OS not reached in the durvalumab arm compared to 29.1 months with placebo [16].

More recently, induction immunotherapy combined with chemotherapy followed by concurrent chemoradiotherapy has been investigated as a strategy for bulky locally advanced NSCLC, reflecting the continued expansion of immunotherapy into earlier treatment settings. The molecular complexity of NSCLC, including genomic driver alterations and microsatellite instability (MSI), further shapes immunotherapy outcomes. In a case report by Durán et al. [17], a patient with metastatic lung adenocarcinoma harboring *ARID1A* gene alterations and sporadic MSI, identified via comprehensive liquid biopsy, achieved a 19-month clinical response to nivolumab. A retrospective single-center study by Sciortino et al. used liquid biopsy (Idylla ctKRAS assay) to detect *KRAS* G12C mutations in stage IV NSCLC patients treated with ICIs, finding that in second-line ICI-treated patients, those harboring the *KRAS* G12C mutation showed significantly longer median PFS (23 months versus 5 months; HR = 3.28; *p* = 0.03) [18]. Immunotherapy resistance driven by specific co-mutations, including in *KEAP1*, *SMARCA4*, and *PTEN*, represents a major emerging challenge, as these alterations shape the tumor microenvironment in ways that attenuate ICI efficacy through immunosuppressive signaling, and their detection via liquid biopsy may help anticipate primary resistance before treatment initiation [19].

Comprehensive reviews confirm that the therapeutic landscape for metastatic NSCLC continues to evolve rapidly with combination strategies and novel biomarker-driven patient selection approaches improving outcomes across histological subtypes [20]. Despite these advances, a substantial proportion of patients fail to derive durable benefit, underscoring the urgent need for refined patient selection strategies and novel predictive biomarkers.

### 3.3. PD-L1 in Tissue (Lung Cancer)

Programmed death-ligand 1 (PD-L1) expression assessed by immunohistochemistry (IHC) on tumor tissue remains the only biomarker currently approved and recommended by the National Comprehensive Cancer Network (NCCN) for guiding immunotherapy decisions in metastatic NSCLC. Four primary antibody–platform combinations are in clinical use: 22C3 and 28-8 on the Dako platform, and SP142 and SP263 on the Ventana platform, each with distinct scoring criteria, thresholds, and corresponding approved drugs, creating a complex and at times inconsistent diagnostic landscape. A comprehensive review of PD-L1 detection methodologies and scoring systems highlighted that the lack of standardization across assays, platforms, and cut-off thresholds remains one of the most significant practical barriers to consistent clinical use with inter-assay concordance varying substantially depending on the cellular compartment scored and the antibody clone employed [21].

A critical challenge lies in the significant intratumoral heterogeneity of PD-L1 expression: approximately 26 to 40% of NSCLC patients exhibit significantly different PD-L1 expression scores in different regions of the same tumor, and a study involving 160 patients demonstrated an approximately 48% difference in PD-L1 expression between surgical resection specimens and matched biopsy specimens. As noted by Hofman et al. [22], the extensive heterogeneity of PD-L1 expression, together with the dynamic nature of its regulation by treatment modalities, the tumor microenvironment, and inflammatory responses, calls for repeated evaluations that tissue biopsy alone cannot practically provide. Furthermore, the extensive N-glycosylation of the PD-L1 protein can impair antibody recognition, potentially generating false-negative results with conventional IHC. The clinical implications of PD-L1 IHC scoring are also subtype-dependent: in contrast to adenocarcinoma, PD-L1 expression has not been found to be significantly associated with immunotherapy efficacy in squamous cell carcinoma and SCLC to date. Importantly, in the study by Moran et al. [23], primary tumor PD-L1 IHC scores were available in fewer than half of patients and did not significantly predict PFS or OS in ICI-treated patients, further supporting the complementary role of liquid biopsy-based PD-L1 monitoring alongside static tissue assessment.

The functional interaction between PD-1 and PD-L1 has been suggested to be an accurate predictor of response to immunotherapy. In advanced NSCLC, one study showed that measuring the actual engagement of the PD-1/PD-L1 axis was strongly associated with clinical benefit from ICI regardless of standard IHC scores. These findings indicate that future biomarker assays may need to shift from purely quantitative assessments toward approaches that capture functional activity, as also highlighted in discussions on biomarker limitations by Tsai et al. [24] and Bodor et al. [25].

### 3.4. PD-L1 in Blood

The limitations of tissue-based PD-L1 assessment, including invasiveness, sampling bias, and inability to capture temporal dynamics, have driven considerable interest in blood-based PD-L1 detection strategies. Liquid biopsy offers multiple analytes through which PD-L1 status can be interrogated, including soluble PD-L1 (sPD-L1), exosomal PD-L1, and PD-L1 expressed on circulating tumor cells (CTCs).

Soluble PD-L1 (sPD-L1) retains the PD-1-binding domain and can competitively inhibit T-cell activation. A systematic review and meta-analysis of blood-based PD-L1 assessment in NSCLC patients undergoing ICI therapy confirmed that higher circulating PD-L1 levels, across multiple analyte types, were associated with inferior survival outcomes, although heterogeneity in assay methods and cut-off values continues to limit cross-study comparability [26]. The prospective study by Moran et al. [23] demonstrated that dynamic changes in PD-L1 expression on blood-based TACs, specifically the upregulation of PD-L1 from pre-treatment (T0) to post-induction (T1), significantly predicted improved progression-free survival (HR, 3.49; 95% CI, 1.5 to 8.3; *p* = 0.0091) and overall survival (HR, 3.058; 95% CI, 1.2 to 7.9; *p* = 0.0410) in NSCLC patients treated with ICIs. Critically, this predictive value was not observed in the non-ICI group, confirming the specificity of the signal for immunotherapy response. PD-L1 expression on CTCs in peripheral blood has been independently associated with worse survival in LC patients: a study by Boffa et al. [27] demonstrated that the cellular expression of PD-L1 in the peripheral blood of LC patients was associated with poorer overall survival, while subsequent work confirmed that PD-L1-positive CTCs at baseline predicted inferior outcomes in patients receiving checkpoint inhibitor therapy, and that conversion to PD-L1-negative status during treatment correlated with clinical benefit (referenced in the broader liquid biopsy biomarker context reviewed by Tamminga et al. and Spagnolo et al. [28,29]. The detection of PD-L1 on CTCs can also serve a dual purpose: beyond predicting ICI response, PD-L1 expression on circulating tumor cells has been shown to correlate with epithelial–mesenchymal transition and poor survival in curatively resected NSCLC.

Exosomal PD-L1 has attracted particular attention as a dynamic and stable liquid biopsy analyte with higher concentrations and greater biological stability compared to ctDNA and CTCs. Extracellular vesicles, including exosomes carrying PD-L1 on their surface, represent another important blood-based compartment: these vesicles can suppress T-cell activity at sites distant from the primary tumor, and their measurement in plasma has been proposed as a dynamic readout of immune checkpoint pathway activity that complements both tissue IHC and CTC-based approaches [10]. Despite the promise of these approaches, liquid biopsy PD-L1 assessment is not yet standardized, and the origin of sPD-L1 has not been definitively established, limiting its current clinical applicability as a standalone predictive biomarker.

### 3.5. TMB in Tissue (Lung Cancer)

Tumor mutational burden (TMB), defined as the number of somatic mutations per megabase of sequenced genome, has emerged as a tissue-based biomarker with the potential to predict responses to ICIs. The underlying rationale is that a higher mutational load increases the probability of generating immunogenic neoantigens capable of eliciting anti-tumor T-cell responses.

In NSCLC, tissue-based TMB assessment by comprehensive genomic profiling through next-generation sequencing (NGS) has been investigated across multiple clinical trials. Different studies have shown controversial results regarding TMB, and while some regulatory approvals exist in the USA, TMB is not currently recommended in routine European practice as a standard biomarker of response to immunotherapy. A dedicated critical review of TMB in NSCLC highlighted multiple sources of variability that limit its clinical implementation, including differences in NGS panel size, bioinformatic pipelines, tumor cellularity thresholds, and the absence of a universally agreed cut-off value, concluding that TMB should currently be interpreted in the context of other biomarkers rather than used in isolation [30]. A study of NSCLC patients treated with ICIs found that when PD-L1 or TMB was used alone as a predictor, the clinical benefit rate was similar (35.3% vs. 29.4%); however, this rate increased to 50.0% when combined. Beyond NSCLC, TMB has been explored as a biomarker in SCLC, where predictive markers remain poorly defined: emerging studies suggest that high TMB may correlate with ICI benefit in SCLC, though tissue scarcity and the biological complexity of this histotype make standardized assessment particularly challenging [31,32]. Recent comprehensive reviews of biomarkers for predicting ICI efficacy in NSCLC have confirmed that a multiparametric approach combining TMB, PD-L1 expression, tumor microenvironment characteristics, and liquid biopsy-derived analytes is likely to provide superior predictive accuracy compared to any single biomarker considered in isolation [33,34].

Despite enthusiasm, tissue TMB requires standardized NGS panels and bioinformatic pipelines, and it suffers from the same spatial sampling limitations as other tissue biomarkers.

### 3.6. Predictive Value of Blood-Based TMB

The analysis of the selected literature identified blood-based TMB (bTMB) as a non-invasive alternative to tissue TMB, leveraging ctDNA analysis to estimate the mutational load from peripheral blood samples. This approach addresses the practical limitations of tissue availability and tumor heterogeneity while enabling serial monitoring. ctDNA has been established as a highly versatile liquid biopsy analyte, with clinical applications spanning early detection, therapy monitoring, residual disease assessment, and resistance profiling, making it the most extensively validated blood-based biomarker in LC molecular oncology [14,35].

Tumor mutation burden has been analyzed through a blood-based assay in a subset of the POPLAR and OAK cases, and blood TMB was found to be a predictive biomarker for progression-free survival in patients receiving atezolizumab in NSCLC [36]. The NILE study further supported blood-based genomic profiling in NSCLC, demonstrating that ctDNA testing showed a 48% increase in the rate of biomarker detection compared to tissue analysis alone with a faster turnaround time. However, bTMB has also inherent limitations: the fraction of ctDNA in plasma varies considerably depending on the tumor stage, location, vascularity, and treatment history, and low ctDNA shedding tumors may yield falsely low bTMB values. In patients with low tumor burden or characterized by low ctDNA shedding, false negative results may occur due to insufficient amounts of detectable tumor-derived DNA in plasma [11,13,14,36,37]. Several designs have been proposed in order to overcome these limitations. First, serial plasma sampling could improve analytical sensitivity compared with single-time point analyses by improving the probability of transient ctDNA release detection during cancer evolution or treatment [13,38,39,40,41]. Second, the application of sensitive technologies, such as optimized NGS panels and ultrasensitive assays, may enhance the detection of low-frequencies variants [7,14,15,36]. In addition, the integration of ctDNA data with radiological, clinical and circulation biomarkers may improve treatment-response assessment and disease monitoring [29,42]. Another point to take into consideration is the clonal hematopoiesis of indeterminate potential (CHIP), which may lead to a misinterpretation of low VAF mutations. This issue may be mitigated by using a paired sequencing of PBMCs DNA by using dedicated bioinformatic filters [36].

In SCLC, where tissue is frequently insufficient and rebiopsy is impractical, the development of bTMB and other ctDNA-derived indices is particularly important: emerging studies have investigated liquid biopsy as a tool to predict immunotherapy efficacy in extensive-stage SCLC, demonstrating that both ctDNA-based mutation profiling and ctDNA clearance dynamics during treatment correlate with clinical outcomes [31,37].

The prognostic and predictive utility of serial ctDNA monitoring has been further demonstrated in NSCLC patients treated with targeted therapies and ICIs: early on-treatment ctDNA dynamics have been shown to predict response and resistance independently of radiological assessment with molecular progression in ctDNA often preceding radiographic progression by weeks to months.

### 3.7. Comparison of ctDNA in Blood vs. Tissue

The integration of liquid biopsy into clinical practice necessitates a rigorous comparison with the current “gold standard”. Our analysis of the selected literature highlights how both the challenges of concordance and the complementary nature between ctDNA-based liquid biopsy and tissue biopsy represent a central question in the clinical implementation of molecular profiling for LC [17] (Table 1).

While tissue remains the gold standard for initial diagnosis, liquid biopsy offers unique advantages in capturing clonal heterogeneity and temporal evolution of the tumor genome. Liquid biopsy could provide a source of information on the resistance mutations of the entire tumor landscape compared with the single site sampled using conventional tumor tissue biopsy [40]. A limitation of liquid biopsy is its inability to reliably discriminate between clonal hematopoiesis-derived variants from tumor-derived alterations. The hematopoietic stem cells acquire somatic mutations, causing a clonal subpopulation of blood cells over time, that characterizes an age-related condition called Clonal Hematopoiesis of Indeterminate Potential (CHIP). The hematopoietic signals may confound genomic analyses. This issue can be mitigated by parallel sequencing of peripheral blood mononuclear cells (PBMCs).

In the context of EGFR-mutant NSCLC, the management of acquired resistance has been substantially informed by liquid biopsy: serial ctDNA monitoring can detect the emergence of resistance mechanisms, including T790M mutations and other bypass pathway activations, often before clinical or radiological progression, enabling pre-emptive therapeutic adaptation [43,45]. In the context of EGFR-mutant NSCLC more broadly, the high specificity (80–95%) of ctDNA testing for driver mutations has been documented, though sensitivity varies from 60% to 85%, and a negative result does not rule out the presence of an oncogenic alteration.

In SCLC, where repeated tissue sampling is particularly challenging due to limited biopsy accessibility and rapidly evolving disease, the comparison of ctDNA with tissue-derived molecular data has highlighted that combined analysis captures a broader spectrum of actionable alterations and resistance mutations than either approach alone [2,37,44].

The perioperative setting represents an important emerging application of liquid biopsy complementarity: recent data from ctDNA analyses in resectable thoracic malignancies demonstrate that post-operative ctDNA detection correlates with minimal residual disease and subsequent relapse, and that ctDNA clearance following neoadjuvant therapy reflects pathological response more dynamically than static tissue assessment alone [46,47]. Crucially, serial liquid biopsy enables the tracking of molecular evolution in ways that repeated tissue sampling cannot practically achieve.

### 3.8. Circulating Immune Cells

Circulating immune cells represent a readily accessible and biologically informative liquid biopsy compartment for interrogating the systemic immune environment in LC patients undergoing immunotherapy. Among these, tumor-associated macrophages (TAMs), T-cell subsets, and circulating tumor cells (CTCs) bearing immune checkpoint molecules have attracted the most attention. In the tumor microenvironment, tumor-associated macrophages are the dominant leukocyte population, and they contribute to tumorigenesis by inhibiting anti-tumor immune surveillance through the release of various immune-mediators.

CTCs in NSCLC have been shown to carry independent prognostic information: higher CTC counts at baseline have been associated with worse tumor response to checkpoint inhibitors, and dynamic changes in CTC burden during treatment have been proposed as an early on-treatment monitoring tool that reflects immunological engagement of the tumor [28,41]. A distinct and particularly novel circulating cell subtype has been characterized by Manjunath et al. [48]: tumor-macrophage fusion (TMF) cells, which are defined as large (≥30 µm) circulating cells co-expressing epithelial/tumor markers (CK/EpCAM+) and myeloid markers (CD14/CD45+). In a prospective, bi-institutional study of 221 individuals, TMF cells were detected in 81% of non-screened NSCLC patients but in only 5.4% of high-risk smoking controls with no or benign-appearing lung nodules (Lung-RADS 1–3), and they were significantly more prevalent in biopsy-proven NSCLC patients within the Lung-RADS 4 suspicious nodule cohort (65.2% versus 14.3% in benign nodule patients; *p* < 0.005) [47]. In SCLC, the enumeration and molecular characterization of CTCs has been investigated as a means of identifying predictors of immunotherapy response: circulating tumor cells in SCLC express surface markers including DLL3, PD-L1, and SLFN11 that may stratify patients for specific therapeutic approaches, and serial CTC monitoring can capture phenotypic evolution during treatment in a setting where repeated tissue biopsy is often impractical [31,44,49].

A broader framework for understanding the clinical utility of circulating biomarkers, including CTCs, CTC clusters, and fusion cells, in the context of treatment response and resistance has been articulated in recent reviews, which emphasize that whole-cell circulating biomarkers provide unique biological information about viable tumor cells and their interactions with the immune system that cell-free analytes such as ctDNA cannot capture [50].

### 3.9. Cytokines

Cytokines, as soluble mediators of immune communication, reflect the functional state of both the tumor microenvironment and the systemic immune response, making their measurement in peripheral blood a potentially valuable addition for immunotherapy monitoring in LC. Among the most studied are GM-CSF (granulocyte-macrophage colony-stimulating factor) and M-CSF (macrophage colony-stimulating factor), which play pivotal roles in the maturation of monocytes into tumor-associated macrophages. Anti-TAM strategies targeting the CSF-1 receptor have demonstrated significant reductions in TAMs in on-treatment tumor biopsies. A systematic review of soluble immunological biomarkers in advanced NSCLC confirmed that a broad array of circulating protein, including cytokines, soluble checkpoint molecules, and immune mediators, show associations with ICI response and survival outcomes, though methodological heterogeneity across studies has so far precluded the validation of any single soluble marker for routine clinical use [51,52].

In the context of immunotherapy response, a transcriptomic analysis of immune markers in tumor tissue can complement blood-based cytokine assessments: RNA-seq data from the metastatic lesion at progression revealed a high expression of CXCL9, indicating activation of the interferon-gamma pathway, a positive predictive marker, despite eventual resistance [17]. In addition, angiopoietin-2 (ANGPT2), a cytokine related to angiogenesis and immune regulation, has been investigated as a circulating biomarker in the immunotherapy setting with high pre-treatment ANGPT2 concentrations associated with reduced response rates. Novel blood-based biomarker strategies for immunotherapy monitoring are also being explored in specific treatment combinations: a study in patients with metastatic oligoprogressive LC treated with stereotactic ablative radiotherapy and immunotherapy investigated novel blood biomarkers for response prediction and monitoring, identifying several soluble immune mediators whose early changes correlated with clinical outcomes, suggesting that combination treatment modalities may induce distinct systemic cytokine signatures amenable to liquid biopsy capture [53].

The cytokine milieu is also shaped by oncogenic driver mutations: in KRAS-mutated NSCLC, activation of the MAPK and AKT-mTOR downstream pathways drives elevated PD-L1 expression and increased leukocyte infiltration [18]. Serum proteomic profiling by mass spectrometry has emerged as a complementary approach for identifying cytokine and protein signatures associated with ICI response and toxicity: a study using mass spectrometry-based serum proteomics in cancer patients treated with immunotherapy identified distinct proteomic signatures predictive of clinical outcomes, including immune-related adverse events, providing a hypothesis-free complement to targeted cytokine assays [42]. The ECM remodeling biomarker VICM (MMP-degraded and citrullinated vimentin), a surrogate of macrophage-mediated tissue turnover, has been proposed as a liquid biopsy tool to monitor TAM activity and predict response to anti-TAM therapies and immunotherapy with data from inflammatory disease models supporting its sensitivity to pharmacodynamic changes in macrophage activation [54]. TMF cells, whose formation from tumor-macrophage fusion events is regulated by local immune and inflammatory signals, may themselves serve as a proxy for macrophage-mediated immunosuppressive cytokine activity in the tumor microenvironment: TMF cell counts are significantly elevated in NSCLC patients and correlate with poor prognosis, suggesting they reflect an active immunosuppressive macrophage phenotype. MicroRNAs represent a further class of circulating immune mediators with biomarker potential in lung cancer: circulating microRNAs have been detected in plasma and serum at altered levels in NSCLC patients, and specific microRNA signatures have been associated with both tumor presence and response to therapy with their stability in blood making them attractive analytes for longitudinal liquid biopsy monitoring [55,56].

A key limitation of cytokine-based biomarkers in the context of ICI monitoring is their inherent lack of tumor specificity. Circulating levels of IL-6, IL-8, IL-10, IFN-γ, and TGF-β are subject to significant confounding by systemic inflammatory conditions, intercurrent infections, smoking status, chronic obstructive pulmonary disease, and metabolic comorbidities—all of which are highly prevalent in the NSCLC patient population [24,52]. Ancel et al. (2023) [52] explicitly noted that the interpretation of soluble biomarkers in NSCLC is complicated by inter-patient biological variability and the absence of standardized pre-analytical protocols, including differences in sample collection timing, storage conditions, and assay platforms. Similarly, Zafra et al. [53] highlighted that cytokine signatures obtained in the setting of combined radiotherapy and immunotherapy may reflect radiation-induced systemic inflammation rather than tumor-intrinsic immune modulation, further complicating their interpretation as predictive biomarkers. Spagnolo et al. [29] further emphasized that the clinical translation of circulating biomarkers in NSCLC, including cytokines, is currently hampered by the lack of prospective validation in homogeneous, well-characterized patient cohorts, and by the confounding effect of performance status, steroid use, and prior lines of therapy on immune cell and cytokine profiles. Tsai et al. (2024) [24] additionally underscored that many cytokine studies in the ICI setting are retrospective, use heterogeneous patient populations, and lack correction for key confounders such as smoking history and the presence of autoimmune comorbidities, which may independently alter baseline cytokine milieu.

In light of these limitations, cytokine profiling should not be interpreted in isolation. Its clinical value is maximized when integrated within multi-analyte platforms that combine ctDNA dynamics, CTC enumeration and phenotyping, and peripheral immune cell profiling alongside clinical and radiological data. Future prospective studies should incorporate standardized pre-analytical workflows, define appropriate patient stratification criteria—including smoking status, performance score, and comorbidity burden—and apply multivariate modeling to disentangle tumor-driven cytokine signals from systemic inflammatory background noise.

The integration of cytokine profiling with other liquid biopsy analytes and imaging biomarkers is expected to refine patient stratification and treatment monitoring as immunotherapy continues to evolve. In the future, the medical digital twin paradigm proposed by Sadée et al. [57] envisions the fusion of cytokine and proteomic data streams alongside genomic and imaging inputs within the patient-in-silico model, enabling dynamic simulation of the tumor-immune interface and the prediction of treatment response across successive therapeutic cycles, which is a vision that positions multi-analyte liquid biopsy, including cytokine profiling, as a central input to next-generation personalized oncology platforms.

## 4. Conclusions and Future Perspectives

The evolution of immunotherapy in NSCLC and SCLC has introduced more complex strategies. Although tissue investigations, such as IHC for PD-L1 and TMB, have represented a starting point for patient selection, as demonstrated by pivotal trials such as KEYNOTE-024 and PACIFIC, they continue to be “snapshot” representations of a highly dynamic disease. The evidence, summarized in this review, suggests that liquid biopsy is evolving from a complementary tool to a central pillar of immuno-oncology.

One of the most significant clinical challenges is the intratumoral heterogeneity of PD-L1 expression. With discordance rates reaching 40% within the same tumor, a single fine-needle biopsy can lead to a false-negative result or an underestimation of the score, potentially depriving patients of potentially beneficial ICI therapy.

The use of longitudinal liquid biopsy, and therefore the study of analytes such as soluble PD-L1 (sPD-L1) and exosomal PD-L1, offers a systemic analysis that integrates signals from multiple metastatic sites.

Unlike IHC, which assesses only protein presence, functional tests and tumor-associated cell (TAC) monitoring reflect activation of the PD-1/PD-L1 axis. The predictive power of increased PD-L1 expression on TACs after induction therapy [23] highlights that the induction of an immune response is clinically more relevant than baseline expression, thus allowing for greater therapeutic accuracy.

Although TMB has encountered obstacles in European clinical practice due to a lack of standardization, blood-based TMB (bTMB) offers a non-invasive solution to the problem of tissue exhaustion, especially in SCLC.

Furthermore, liquid biopsy allows for an early prediction of treatment response. Indeed, in addition to a static baseline count, ctDNA shedding dynamics have emerged as a “molecular RECIST.” Molecular progression often precedes radiographic progression by months, allowing for a “lead time” during which clinicians could theoretically switch therapy before the patient experiences symptomatic worsening.

The identification of co-mutations such as KEAP1, STK11, and PTEN via liquid biopsy provides a deeper understanding of primary resistance. These alterations shape a “cold” tumor microenvironment that PD-L1 expression alone cannot explain, underscoring the need for the multiparametric approach suggested by Zhang et al. [33].

A recent research area of particular interest is the study of the systemic immune environment through tumor–macrophage fusion (TMF) cells and cytokine profiling. In fact, the presence of TMF cells (CD14+/CK+) directly reflects the immunosuppressive activity of tumor-associated macrophages. These cells are not only markers of presence but also represent functional units of the “pre-metastatic niche.” Regarding cytokine profiles, the systemic “cytokine storm” or specific signatures (e.g., ANGPT2 or CXCL9) provide information on the interferon-gamma pathway. Integrating these proteomic signals with genomic data (ctDNA) creates a holistic view of the tumor-immunity cycle. Although prospective head-to-head correlative analyses between tissue PD-L1 expression and blood-based biomarkers within the same patient cohorts remain limited, several original studies have explored this relationship. A concordance between PD-L1 expression measured on CTCs and on matched tumor biopsies has been reported by Moran et al. (2022) [23], supporting CTC-based PD-L1 assessment as a dynamic surrogate for tumor PD-L1 status. Boffa et al. [27] demonstrated that peripheral blood PD-L1 expression correlates with worse survival outcomes in NSCLC, establishing an independent prognostic value for blood-based PD-L1 measurement. With respect to bTMB, partial but imperfect concordance with tissue TMB has been reported [30], reflecting biological differences between circulating and tissue-derived DNA. Proteomic serum signatures, as described by Park et al. (2022) [42], have also shown potential to predict ICI response, further supporting a multi-analyte blood-based approach. The field would benefit substantially from prospective studies that simultaneously assess tissue PD-L1, bTMB, ctDNA dynamics, CTC-derived PD-L1, and cytokine profiles within the same patient cohort in order to establish integrated multi-omic predictive models for ICI response in NSCLC, which is a priority direction for future research in this area.

Among currently available liquid biopsy biomarkers, ctDNA profiling represents the most clinically mature approach [7,14,36,38]. bTMB also represents a promising strategy for immunotherapy stratification, although its broader implementation is still limited by technical variability and lack of standardized cut-offs [30,34]. Differently, PDL-1 expression on CTCs, extracellular vesicles and soluble PDL-1 remains investigational because of heterogeneous methodologies and insufficient prospective validation [24,26,29]. Comparably, CTCs, cytokines, proteomic profiles and miRNA base biomarkers are limited by a lack of assay standardization and a heterogenous validation cohort, which remains to be implemented into clinical routine [29,41,42,44]. While several biomarkers’ classes are promising, further larger-scale prospective studies and harmonized analytical workflows are required before broad clinical adoption.

Despite the clear biological advantages, several obstacles remain before liquid biopsy can fully replace tissue biopsies for immunotherapy targeting. Standardization is still lacking. The lack of uniformity between platforms (Dako vs. Ventana) and bioinformatics pipelines for transmembrane biopsy (bTMB) remains a hurdle. Sensitivity in patients with low ctDNA release or localized disease may present false negatives, necessitating a “tissue first, then liquid biopsy” or “parallel” approach [21]. However, a digital future is emerging where the proposed medical digital twin paradigm [54] offers a solution to this complexity. By integrating real-time liquid biopsy data into in silico models, we can overcome population-based thresholds in favor of dynamic, personalized probability scores for treatment success.

In conclusion, liquid biopsy provides a unique, real-time molecular picture of the tumor-immune landscape, which tissue biopsy is virtually incapable of achieving. While tissue remains essential for initial histological classification, the longitudinal monitoring of ctDNA, CTCs, and soluble molecules enables a truly adaptive therapeutic strategy. The studies reviewed here are predominantly observational, retrospective or proof-of-concept in design, and currently, large randomized trials with survival as a primary endpoint are lacking. Liquid biopsy should therefore be considered as a complementary tool to standard ones rather than a standalone replacement with the hope that ongoing prospective trials will define its clinical utility. Liquid biopsy is emerging as a transformative tool in the management of NSCLC patients receiving immune checkpoint inhibitor therapy. The analytes reviewed herein—ctDNA, bTMB, CTCs, exosomes, soluble PD-L1, circulating immune cells, and cytokines—collectively offer a multi-dimensional, real-time portrait of tumor dynamics and immune contexture that cannot be obtained from a single tumor biopsy.

From a mechanistic standpoint, ctDNA levels reflect overall tumor burden and genomic instability, both of which are tightly linked to the immunogenic neoantigen landscape of the tumor. High somatic mutational load, as captured by bTMB, correlates with increased neoantigen presentation on MHC class I molecules, which in turn promotes CD8+ cytotoxic T lymphocyte (CTL) priming and activation [25,30]. Activated CTLs release IFN-γ, which upregulates PD-L1 expression on tumor cells via the JAK1/JAK2–STAT1 signaling axis, representing the canonical adaptive immune resistance mechanism [24]. Thus, elevated bTMB may paradoxically predict both greater immunogenicity and a higher degree of PD-L1-mediated immune evasion, explaining why ICI therapy is particularly effective in this subgroup. The dynamic monitoring of ctDNA during treatment provides an integrated, real-time readout of tumor burden reduction and clonal evolution under immune pressure, anticipating radiological response by several weeks [38,39].

The standardization of pre-analytical and analytical protocols, validation in prospective multicenter cohorts, and integration of multi-omic data through machine learning approaches represent the critical next steps toward clinical implementation. The convergence of these technologies holds the promise of truly personalized immuno-oncology, in which treatment decisions are continuously informed by minimally invasive blood-based monitoring.

The future of lung cancer immunotherapy will likely rely on a unified diagnostic workflow in which the molecular pathologist integrates data from both tissue and fluids to navigate the complexities of tumor evolution and resistance.

## Figures and Tables

**Table 1:** Advantages and limitations of liquid biopsy in lung cancer.

Aspect	Benefits	Limitations	Clinical Impact	References
Invasiveness	Minimally invasive	Cannot fully replace tissue biopsy in all cases	Useful when tissue is unavailable or insufficient	[7,8,11,12,14,15]
Sensitivity	ability in detecting tumor-derived alterations in high-shedding tumors	Reduced sensitivity in low tumor burden or low-shedding tumors	Risk of false negatives; negative results may require tissue confirmation	[7,11,13,14,36,39]
Specificity	High specificity with advanced NGS approaches	Confounding by clonal hematopoiesis (CHIP)	Requires careful interpretation and validation on PBMCs	[7,12,14,15,22]
Tumour Heterogeneity	Captures spatial and temporal heterogeneity better than single-site biopsy	May not detect alterations from non-shedding clones	Complements tissue biopsy for a more comprehensive genomic profile	[5,7,11,13,14,15]
Dynamic monitoring	Enables real-time assessment of treatment response and resistance	Biological variability in ctDNA levels	Optimal timing and longitudinal sampling strategies are critical	[13,14,36,39,40,41]
Turnaround time	Faster than tissue-based genomic profiling	Dependent on platform and infrastructure availability	Facilitates rapid treatment decisions in advanced disease	[7,11,12,15]
Accessibility and repeatibility	Easily repeatable over time	Limited standardization across platforms and assays	Need for harmonization of protocols for clinical implementation	[7,8,11,14,15]
Detection of resistance mechanisms	Early identification of resistance mutations	May miss resistance driven by non-genomic mechanisms	Should be integrated with clinical and radiological data	[13,36,39,41,43,44]

## Data Availability

All the data used in this review are findable by applying the literature search shown in Figure 1.

## Abbreviations

The following abbreviations are used in this manuscript:

bTMB	blood-based Tumor Mutational Burden
CHIP	Clonal Hematopoiesis of Indeterminate Potential
CSF	Cerebrospinal Fluid
ctDNA	Circulating Tumor DNA
CTC	Circulating Tumor Cells
ECM	Extracellular Matrix
IASLC	International Association for the Study of Lung Cancer
ICI	Immune Checkpoint Inhibitor
IHC	Immunohistochemistry
LC	Lung Cancer
MSI	Microsatellite Instability
NGS	Next-Generation Sequencing
NCCN	National Comprehensive Cancer Network
NSCLC	Non-Small-Cell Lung Cancer
OS	Overall Survival
PFS	Progression-Free Survival
SCLC	Small-Cell Lung Cancer
sPD-L1	Soluble PD-L1
TAC	Tumor-associated cell
TAM	Tumor-associated macrophages
TMB	Tumor Mutational Burden
VICM	MMP-Degraded And Citrullinated Vimentin

## References

[1] Zhang J., Song Z., Zhang Y., Zhang C., Xue Q., Zhang G., Tan F. (2025). Recent advances in biomarkers for predicting the efficacy of immunotherapy in non-small cell lung cancer. Front. Immunol..

[2] Santamaria S., Cardinali B., Rovere M., Marconi S., Nardin S., Sacco G., Coco S. (2025). New insight in early detection and precision medicine in small cell lung cancer: Liquid biopsy as innovative clinical tool. Crit. Rev. Clin. Lab. Sci..

[3] Cai L.L., Wang J. (2019). Liquid biopsy for lung cancer immunotherapy. Oncol. Lett..

[4] Brozos-Vázquez E.M., Díaz-Peña R., García-González J., León-Mateos L., Mondelo-Macía P., Peña-Chilet M., López-López R. (2021). Immunotherapy in non-small-cell lung cancer: Current status and future prospects for liquid biopsy. Cancer Immunol. Immunother..

[5] Yang Y., Liu H., Chen Y., Xiao N., Zheng Z., Liu H., Wan J. (2023). Liquid biopsy on the horizon in immunotherapy of non-small cell lung cancer: Current status, challenges, and perspectives. Cell Death Dis..

[6] Deshpande R., Chandra M., Rauthan A. (2022). Evolving trends in lung cancer: Epidemiology, diagnosis, and management. Indian J. Cancer.

[7] Rolfo C., Mack P., Scagliotti G.V., Aggarwal C., Arcila M.E., Barlesi F., Bivona T., Diehn M., Dive C., Dziadziuszko R. (2021). Liquid biopsy for advanced NSCLC: A consensus statement from the International Association for the Study of Lung Cancer. J. Thorac. Oncol..

[8] Hofman P., Heeke S., Alix-Panabières C., Pantel K. (2019). Liquid biopsy in the era of immuno-oncology: Is it ready for prime-time use for cancer patients?. Ann. Oncol..

[9] Liu Y., Wang S., Xia H., Tan X., Song S., Zhang S., Meng D., Chen Q., Jin Y. (2022). The potential applications of microparticles in the diagnosis, treatment, and prognosis of lung cancer. J. Transl. Med..

[10] Bandini S., Ulivi P., Rossi T. (2024). Extracellular vesicles, circulating tumor cells, and immune checkpoint inhibitors: Hints and promises. Cells.

[11] Bonanno L., Dal Maso A., Pavan A., Zulato E., Calvetti L., Pasello G., Guarneri V., Conte P., Indraccolo S. (2022). Liquid biopsy and non-small cell lung cancer: Are we looking at the tip of the iceberg?. Br. J. Cancer.

[12] Bertoli E., De Carlo E., Basile D., Zara D., Stanzione B., Schiappacassi M., Del Conte A., Spina M., Bearz A. (2023). Liquid biopsy in NSCLC: An investigation with multiple clinical implications. Int. J. Mol. Sci..

[13] Kilgour E., Rothwell D.G., Brady G., Dive C. (2020). Liquid biopsy-based biomarkers of treatment response and resistance. Cancer Cell.

[14] Aredo J.V., Jamali A., Zhu J., Heater N., Wakelee H.A., Vaklavas C., Anagnostou V., Lu J. (2025). Liquid biopsy approaches for cancer characterization, residual disease detection, and therapy monitoring. ASCO Educ. Book.

[15] Kontic M., Stjepanovic M., Markovic F. (2025). Beyond the tissue: Unlocking NSCLC treatment potential through liquid biopsy. Genes.

[16] Alexander M., Kim S.Y., Cheng H. (2020). Update 2020: Management of non-small cell lung cancer. Lung.

[17] Durán M., Faull I., Lastra E., Laes J.F., Rodrigo A.B., Sánchez-Escribano R. (2021). *ARID1A* genomic alterations driving microsatellite instability through somatic MLH1 methylation with response to immunotherapy in metastatic lung adenocarcinoma: A case report. J. Med. Case Rep..

[18] Sciortino C., Viglialoro V., Nucci M., Polito M.G., Cortesi E., Gelibter A., Gazzaniga P., Nicolazzo C., Siringo M., Caponnetto S. (2022). Response to immunotherapy in KRAS G12C mutated NSCLC: A single-centre retrospective observational study. Oncotarget.

[19] Paredes R., Borea R., Drago F., Russo A., Nigita G., Rolfo C. (2025). Genetic drivers of tumor microenvironment and immunotherapy resistance in non-small cell lung cancer: The role of *KEAP1*, *SMARCA4*, and *PTEN* mutations. J. Immunother. Cancer.

[20] Russano M., La Cava G., Cortellini A., Citarella F., Galletti A., Di Fazio G.R., Santo V., Brunetti L., Vendittelli A., Fioroni I. (2023). Immunotherapy for metastatic non-small cell lung cancer: Therapeutic advances and biomarkers. Curr. Oncol..

[21] Qi C., Li Y., Zeng H., Wei Q., Tan S., Zhang Y., Li W., Tian P. (2024). Current status and progress of PD-L1 detection: Guiding immunotherapy for non-small cell lung cancer. Clin. Exp. Med..

[22] Hofman P., Berezowska S., Kazdal D., Mograbi B., Ilié M., Stenzinger A., Hofman V. (2024). Current challenges and practical aspects of molecular pathology for non-small cell lung cancers. Virchows Arch..

[23] Moran J.A., Adams D.L., Edelman M.J., Lopez P., He J., Qiao Y., Xu T., Liao Z., Gardner K.P., Tang C.-M. (2022). Monitoring PD-L1 expression on circulating tumor-associated cells in recurrent metastatic non-small-cell lung carcinoma predicts response to immunotherapy with radiation therapy. JCO Precis. Oncol..

[24] Tsai Y.T., Schlom J., Donahue R.N. (2024). Blood-based biomarkers in patients with non-small cell lung cancer treated with immune checkpoint blockade. J. Exp. Clin. Cancer Res..

[25] Bodor J.N., Boumber Y., Borghaei H. (2020). Biomarkers for immune checkpoint inhibition in non-small cell lung cancer (NSCLC). Cancer.

[26] Cui Q., Li W., Wang D., Wang S., Yu J. (2023). Prognostic significance of blood-based PD-L1 analysis in patients with non-small cell lung cancer undergoing immune checkpoint inhibitor therapy: A systematic review and meta-analysis. World J. Surg. Oncol..

[27] Boffa D.J., Graf R.P., Salazar M.C., Hoag J., Lu D., Krupa R., Louw J., Dugan L., Wang Y., Landers M. (2017). Cellular expression of PD-L1 in the peripheral blood of lung cancer patients is associated with worse survival. Cancer Epidemiol. Biomark. Prev..

[28] Tamminga M., de Wit S., Hiltermann T.J.N., Timens W., Schuuring E., Terstappen L.W.M.M., Groen H.J.M. (2019). Circulating tumor cells in advanced non-small cell lung cancer patients are associated with worse tumor response to checkpoint inhibitors. J. Immunother. Cancer.

[29] Spagnolo C.C., Pepe F., Ciappina G., Nucera F., Ruggeri P., Squeri A., Speranza D., Silvestris N., Malapelle U., Santarpia M. (2024). Circulating biomarkers as predictors of response to immune checkpoint inhibitors in NSCLC: Are we on the right path?. Crit. Rev. Oncol. Hematol..

[30] Meri-Abad M., Moreno-Manuel A., García S.G., Calabuig-Fariñas S., Pérez R.S., Herrero C.C., Jantus-Lewintre E. (2023). Clinical and technical insights of tumour mutational burden in non-small cell lung cancer. Crit. Rev. Oncol. Hematol..

[31] Fasano R., Serratì S., Rafaschieri T., Longo V., Di Fonte R., Porcelli L., Azzariti A. (2024). Small-cell lung cancer: Is liquid biopsy a new tool able to predict the efficacy of immunotherapy?. Biomolecules.

[32] Lorenzi M., Resi M.V., Bonanno L., Frega S., Dal Maso A., Ferro A., Guarneri V., Pasello G. (2024). Tissue and circulating biomarkers of benefit to immunotherapy in extensive-stage small cell lung cancer patients. Front. Immunol..

[33] Zhang Y.R., Lu Y.H., Lin C.M., Ku J.W. (2025). Pretreatment CT texture analysis for predicting survival outcomes in advanced non-small cell lung cancer patients receiving immunotherapy: A systematic review and meta-analysis. Thorac. Cancer.

[34] Li S., Zhang C., Pang G., Wang P. (2020). Emerging blood-based biomarkers for predicting response to checkpoint immunotherapy in non-small-cell lung cancer. Front. Immunol..

[35] Brozos-Vázquez E.M., Rodríguez-López C., Cortegoso-Mosquera A., López-Landrove S., Muinelo-Romay L., García-González J., López-López R., León-Mateos L. (2023). Immunotherapy in patients with brain metastasis: Advances and challenges for the treatment and the application of circulating biomarkers. Front. Immunol..

[36] Duffy M.J. (2024). Circulating tumor DNA (ctDNA) as a biomarker for lung cancer: Early detection, monitoring and therapy prediction. Tumour Biol..

[37] Galant N., Grenda A., Krawczyk P., Pięt M., Milanowski J. (2025). Liquid biopsy in diagnosis and monitoring of treatment efficacy in patients with small cell lung cancer. Mol. Biol. Rep..

[38] Frank M.S., Andersen C.S.A., Ahlborn L.B., Pallisgaard N., Bodtger U., Gehl J. (2022). Circulating tumor DNA monitoring reveals molecular progression before radiologic progression in a real-life cohort of patients with advanced non-small cell lung cancer. Cancer Res. Commun..

[39] Horndalsveen H., Alver T.N., Dalsgaard A.M., Rogg L.V., Helbekkmo N., Grønberg B.H., Halvorsen T.O., Ramberg C., Haakensen V.D., Öjlert Å.K. (2023). Atezolizumab and stereotactic body radiotherapy in patients with advanced non-small cell lung cancer: Safety, clinical activity and ctDNA responses—The ComIT-1 trial. Mol. Oncol..

[40] Melichar B. (2022). Biomarkers in the management of lung cancer: Changing the practice of thoracic oncology. Clin. Chem. Lab. Med..

[41] Chrzempiec M., Oleksiewicz U. (2025). Circulating tumor cells for the monitoring of lung cancer therapies. Int. J. Mol. Sci..

[42] Park Y., Kim M.J., Choi Y., Kim N.H., Kim L., Hong S.P.D., Cho H.-G., Yu E., Chae Y.K. (2022). Role of mass spectrometry-based serum proteomics signatures in predicting clinical outcomes and toxicity in patients with cancer treated with immunotherapy. J. Immunother. Cancer.

[43] Reckamp K.L., Patil T., Kirtane K., Rich T.A., Espenschied C.R., Weipert C.M., Raymond V.M., Santana-Davila R., Doebele R.C., Baik C.S. (2020). Duration of targeted therapy in patients with advanced non-small-cell lung cancer identified by circulating tumor DNA analysis. Clin. Lung Cancer.

[44] Shrestha P., Kao S., Cheung V.K., Cooper W.A., van Zandwijk N., Rasko J.E.J., Yeo D. (2025). Circulating tumor cells: Advancing personalized therapy in small cell lung cancer patients. Mol. Oncol..

[45] Wu S.G., Shih J.Y. (2018). Management of acquired resistance to EGFR TKI-targeted therapy in advanced non-small cell lung cancer. Mol. Cancer.

[46] Reuss J.E., Lee P.K., Mehran R.J., Hu C., Ke S., Jamali A., Najjar M., Niknafs N., Wehr J., Oner E. (2025). Perioperative nivolumab or nivolumab plus ipilimumab in resectable diffuse pleural mesothelioma: A phase 2 trial and ctDNA analyses. Nat. Med..

[47] Ohara S., Suda K., Tsutani Y. (2025). Utility and future perspectives of circulating tumor DNA analysis in non-small cell lung cancer patients in the era of perioperative chemo-immunotherapy. Cells.

[48] Manjunath Y., Suvilesh K.N., Mitchem J.B., Avella Patino D.M., Kimchi E.T., Staveley-O’Carroll K.F., Pantel K., Yi H., Li G., Harris P.K. (2022). Circulating tumor-macrophage fusion cells and circulating tumor cells complement non-small-cell lung cancer screening in patients with suspicious Lung-RADS 4 nodules. JCO Precis. Oncol..

[49] Ji K., Guo L., Zuo D., Feng M., Chen X., Zhao Z., Tang J., Chen G. (2025). Harnessing delta-like ligand 3: Bridging biomarker discovery to next-generation immunotherapies in refractory small cell lung cancer. Front. Immunol..

[50] Lee E.Y., Kulkarni R.P. (2019). Circulating biomarkers predictive of tumor response to cancer immunotherapy. Expert Rev. Mol. Diagn..

[51] Mildner F., Sopper S., Amann A., Pircher A., Pall G., Köck S., Naismith E., Wolf D., Gamerith G. (2020). Systematic review: Soluble immunological biomarkers in advanced non-small-cell lung cancer (NSCLC). Crit. Rev. Oncol. Hematol..

[52] Ancel J., Dormoy V., Raby B.N., Dalstein V., Durlach A., Dewolf M., Gilles C., Polette M., Deslée G. (2023). Soluble biomarkers to predict clinical outcomes in non-small cell lung cancer treated by immune checkpoint inhibitors. Front. Immunol..

[53] Zafra J., Onieva J.L., Oliver J., Garrido-Barros M., González-Hernández A., Martínez-Gálvez B., Román A., Ordóñez-Marmolejo R., Pérez-Ruiz E., Benítez J.C. (2024). Novel blood biomarkers for response prediction and monitoring of stereotactic ablative radiotherapy and immunotherapy in metastatic oligoprogressive lung cancer. Int. J. Mol. Sci..

[54] Willumsen N., Thomsen L.B., Bager C.L., Jensen C., Karsdal M.A. (2018). Quantification of altered tissue turnover in a liquid biopsy: A proposed precision medicine tool to assess chronic inflammation and desmoplasia associated with a pro-cancerous niche and response to immunotherapeutic modalities. Cancer Immunol. Immunother..

[55] Frydrychowicz M., Kuszel Ł., Dworacki G., Budna-Tukan J. (2023). MicroRNA in lung cancer—A novel potential way for early diagnosis and therapy. J. Appl. Genet..

[56] McCabe M.J., Pinese M., Chan C.-L., Sheriff N., Thompson T.J., Grady J., Wong M., Gauthier M.-E.A., Puttick C., Gayevskiy V. (2019). Genomic stratification and liquid biopsy in a rare adrenocortical carcinoma (ACC) case, with dual lung metastases. Cold Spring Harb. Mol. Case Stud..

[57] Sadée C., Testa S., Barba T., Hartmann K., Schuessler M., Thieme A., Church G.M., Okoye I., Hernandez-Boussard T., Hood L. (2025). Medical digital twins: Enabling precision medicine and medical artificial intelligence. Lancet Digit. Health.

